# Penicillinolide A: A New Anti-Inflammatory Metabolite from the Marine Fungus *Penicillium* sp. SF-5292

**DOI:** 10.3390/md11114510

**Published:** 2013-11-12

**Authors:** Dong-Sung Lee, Wonmin Ko, Tran Hong Quang, Kyoung-Su Kim, Jae Hak Sohn, Jae-Hyuk Jang, Jong Seog Ahn, Youn-Chul Kim, Hyuncheol Oh

**Affiliations:** 1 Department of Pharmacy, College of Pharmacy, Wonkwang University, Iksan 570-749, Korea; E-Mails: hsds@wku.ac.kr (D.-S.L.); rabis@wku.ac.kr (W.K.); quangth2004@yahoo.com (T.H.Q); pipo5@wku.ac.kr (K.-S.K.); 2 Hanbang Body-Fluid Research Center, Wonkwang University, Iksan 570-749, Korea; 3 Standardized Material Bank for New Botanical Drugs, College of Pharmacy, Wonkwang University, Iksan 570-749, Korea; 4 Institute of Marine Biochemistry, Vietnam Academy of Science and Technology (VAST), 18 Hoang Quoc Viet, Caugiay, Hanoi 10000, Vietnam; 5 College of Medical and Life Sciences, Silla University, Busan 617-736, Korea; E-Mail: jhsohn@silla.ac.kr; 6 Chemical Biology Research Center, Korea Research Institute of Bioscience and Biotechnology (KRIBB), 30 Yeongudanji-ro, Ochang, Cheongwon 363-883, Korea; E-Mails: jangjh@kribb.re.kr (J.-H.J.); jsahn@kribb.re.kr (J.S.A.)

**Keywords:** *Penicillium* sp., marine-derived fungi, 10-membered lactone, anti-inflammatory effect, heme oxygenase-1

## Abstract

In the course of studies on bioactive metabolites from marine fungi, a new 10-membered lactone, named penicillinolide A (**1**) was isolated from the organic extract of *Penicillium* sp. SF-5292 as a potential anti-inflammatory compound. The structure of penicillinolide A (**1**) was mainly determined by analysis of NMR and MS data and Mosher’s method. Penicillinolide A (**1**) inhibited the production of NO and PGE_2_ due to inhibition of the expression of iNOS and COX-2. Penicillinolide A (**1**) also reduced TNF-α, IL-1β and IL-6 production, and these anti-inflammatory effects were shown to be correlated with the suppression of the phosphorylation and degradation of IκB-α, NF-κB nuclear translocation, and NF-κB DNA binding activity. In addition, using inhibitor tin protoporphyrin (SnPP), a competitive inhibitor of HO activity, it was verified that the inhibitory effects of compound **1** on the production of pro-inflammatory mediators and NF-κB DNA binding activity were partially associated with HO-1 expression through Nrf2 nuclear translocation.

## 1. Introduction

Prolonged inflammation can lead to a variety of diseases, including arthritis, inflammatory bowel disease, neurodegenerative disorders, and septic shock syndrome. Although the inflammatory responses are different in various diseases, they can be characterized by the involvement of a common spectrum of genes and mediators, including inflammatory cytokines and pro-inflammatory factors [1]. Heme oxygenase-1 (HO-1) is a rate-limiting enzyme in heme catabolism, which leads to the formation of carbon monoxide (CO), iron ions and biliverdin/bilirubin [2]. HO-1 and its by-products play important roles in the resolution phase of inflammation, with macrophages acting as the critical target [3,4]. Studies have shown that HO-1 expression inhibits the production of pro-inflammatory cytokines and chemokines such as tumor necrosis factor (TNF)-α, interleukin (IL)-1β and IL-6 in activated macrophages [5,6,7,8]. Furthermore, the upregulation of HO-1 expression suppresses the expression of the pro-inflammatory cyclooxygenase (COX)-2 and inducible nitric oxide synthase (iNOS), and thereby reduces COX-2-drived prostaglandin E_2_ (PGE_2_) and iNOS-derived nitric oxide (NO) production [9,10,11]. In addition, HO-1 inhibits iNOS expression and NO production in activated macrophages through inactivation of nuclear factor (NF)-κB [10,11,12,13,14]. Thus, a number of therapeutic agents that upregulate the expression of HO-1 and exert anti-inflammatory activities through HO-1 induction have been reported [15,16,17]. Among the various anti-oxidative and anti-inflammatory enzymes, nuclear factor-E2-related factor 2 (Nrf2) plays a key role in the protection of cells against oxidative stress and inflammatory condition [18]. Nuclear translocation of Nrf2 is required for the expression of certain inducible proteins, such as GSH S-transferase, quinine reductase and HO-1 [19]. Recent study has shown that natural products can activate Nrf2 by directly binding to Keap1 through a covalent linkage, which results in the induction of cytoprotective proteins including HO-1 [20]. In addition, our previous studies on the metabolites from marine-derived fungi have resulted in the identification of HO-1 regulating activity and the investigation of the mechanism of the pharmacological activities related to anti-inflammatory activity [21,22].

Fungi have proven to be valuable resources for the discovery of novel secondary metabolites. Because the marine environment provides unique ecosystems and living conditions, marine fungi have been recognized as a potential source of diverse novel secondary metabolites [23,24,25]. In our ongoing studies on bioactive secondary metabolites from marine microorganisms from Korea [21,22,26,27], we investigated the chemical constituents of the extracts obtained from cultures of the marine-derived fungus *Penicillium* sp. SF-5292, which inhibited NO production in LPS-stimulated macrophages. This study led to the isolation of a new 10-membered lactone type metabolite, named penicillinolide A (**1**).

## 2. Results and Discussion

### 2.1. Structure Determination of Penicillinolide A *(1)*

**Figure 1:**
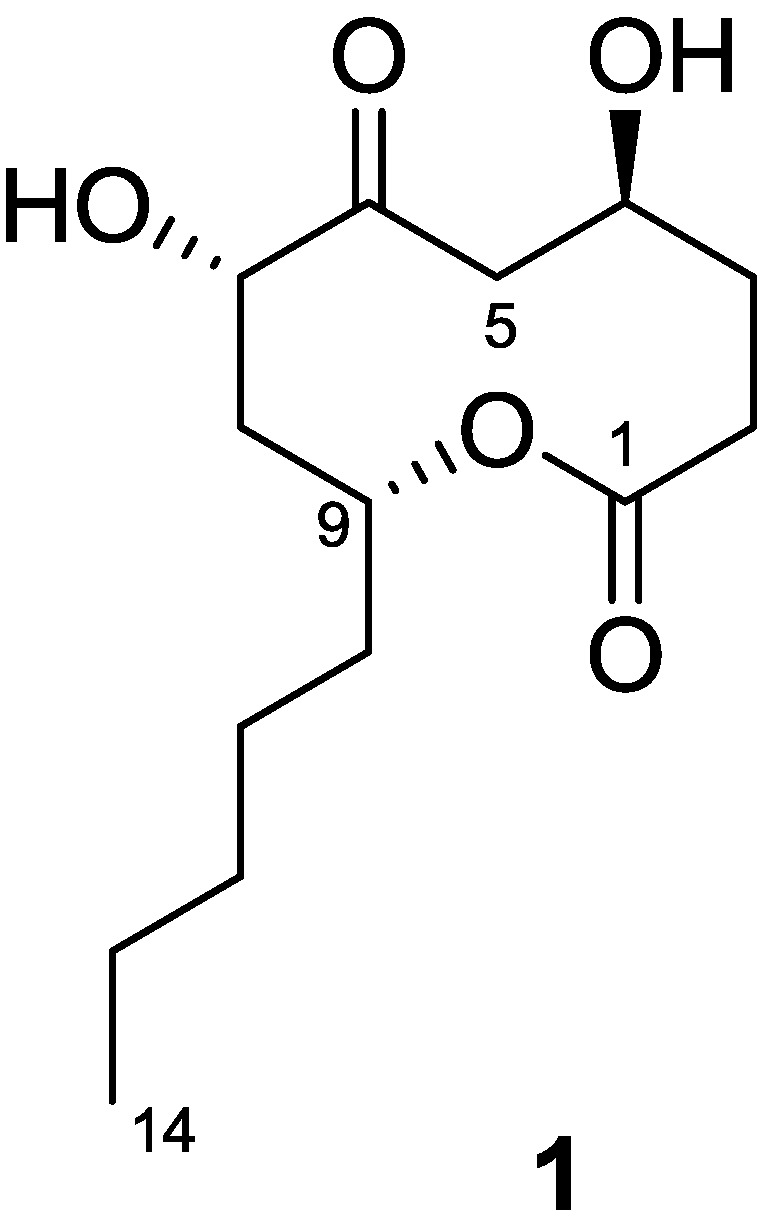
Chemical structure of penicillinolide A (**1**).

Penicillinolide A (**1**) (Figure 1) was assigned the molecular formula C_14_H_24_O_5_ on the basis of HRESIMS data (*m/z* 295.1517 [M + Na]^+^), which was fully supported by the ^1^H and ^13^C NMR data (Table 1). Analysis of ^1^H, ^13^C, and DEPT NMR spectra indicated the presence of one methyl, three oxymethine, and six methylene groups. In addition, the presence of a ketone (δ 211.0) and a carboxylic carbonyl group (δ 172.9) were suggested by the ^13^C NMR spectrum. This structural information accounted for two unsaturation equivalents, suggesting that the compound must be cyclic to account for the unsaturation equivalents required by the molecular formula. In addition, the presence of two hydroxyl groups was suggested by taking into account the molecular formula and chemical shift values for two oxymethine groups (δ 65.2/5.00, δ 75.2/4.57). The presence of a spin system composed of C-2-C-5 was readily identified by analysis of COSY and HSQC data. Another spin system from C-7 to C-11 was also easily identified by analysis of COSY and HSQC data, but further extension of the spin system was hampered by signal overlapping between δ 1.09–1.23. However, observation of HMBC correlation of H-10 with C-11 and C-12, of H-11 with C-12 and C-13, and of H-14 with C-12 and C-13 allowed the completion of the spin system composed of C-8-C-14. Connection of these spin systems and quaternary carbons were established by the observation of key HMBC correlations. Considering the chemical shift values of C-1 (δ 172.9) and C-9 (δ 73.1), HMBC correlation of H-9 with C-1 allowed the connection of C-9 with C-1 via the oxygen atom. The ketone group was attached to C-7 by correlation of H-8 with C-6. HMBC correlation of H-3 with C-5 and of H-5 with C-3, C-4, and C-6 allowed the connection between C-4 and C-5. Therefore, the gross structure of **1** was assigned as shown.

**Figure 2:**
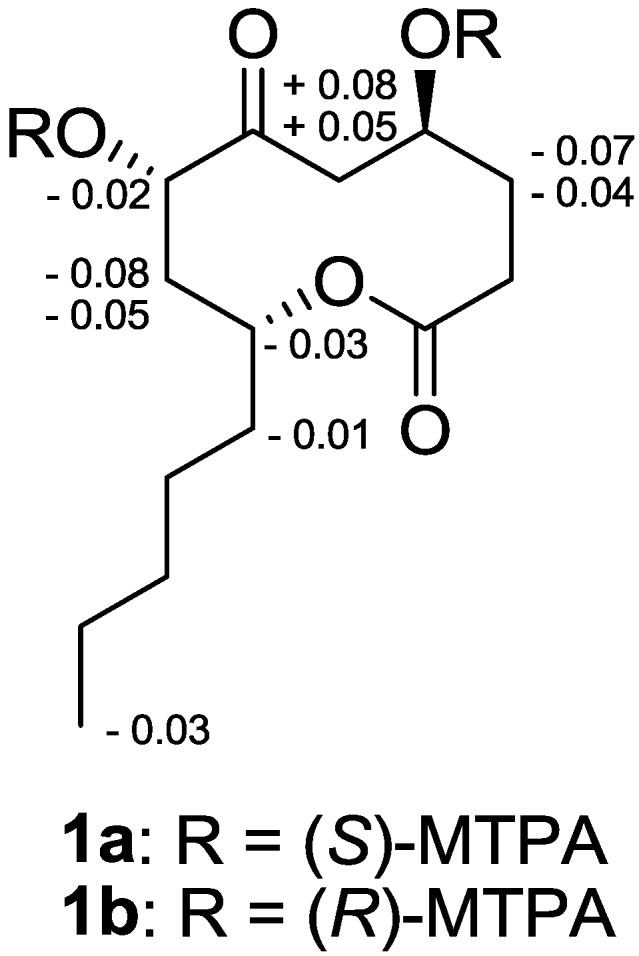
Δδ values [Δδ (in ppm) = δ_S_ − δ_R_] obtained for the (*S*)- and (*R*)-MTPA esters of penicillinolide A (**1a** and **1b**, respectively).

**Figure 3:**
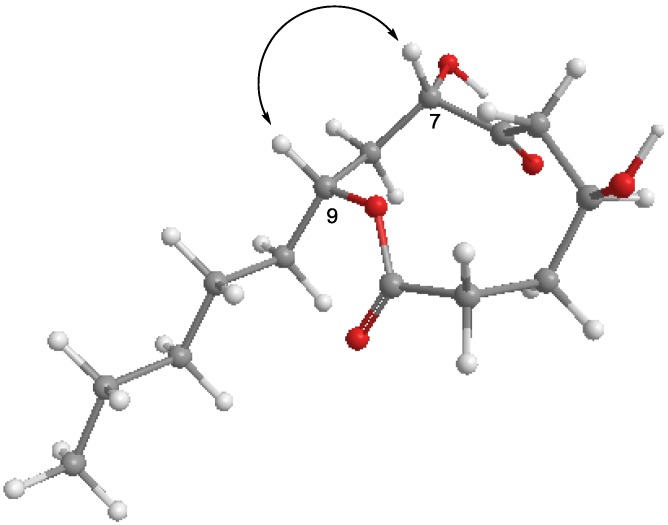
Key NOE correlation of penicillinolide A.

The absolute configuration of **1** was determined by application of modified Mosher’s method. The (*S*)- and (*R*)-MTPA esters of **1** (**1a** and **1b**) were prepared using (*R*)-and (*S*)-MTPA chloride, respectively. Although the complete assignment of protons in **1a** and **1b** was hampered by signal overlapping, careful analysis of the ^1^H NMR and COSY spectra allowed the assignment of the proton chemical shifts for the two diastereomeric esters **1a** and **1b** in proximity of the esterified carbons (C-4 and C-7). The differences in chemical shift values (Δδ =δ_S_ − δ_R_) for the two diastereomeric esters **1a** and **1b** were calculated in order to assign the absolute configurations at C-4 and C-7 (Figure 2). Calculations for all of the relevant signals suggested *S* absolute configurations at C-4 and C-7. With these assignments, NOESY correlations between H-7 and H-9 revealed that these protons are positioned at the same face of the molecule (Figure 3), thus assigning the absolute configuration at C-9 as *R*.

**Table 1:** NMR Spectroscopic Data (^1^H 400 MHz, pyridine-*d*_5_) for Penicillinolide A (**1**).

Position	δ_C_^a^	δ_H_, mult. (*J* in Hz) ^b^	Key NOESY	HMBC (H→C#)
1	172.9	-	-	
2	28.9	3.11, m; 2.56, m	-	1, 3, 4
3	28.6	2.65, m; 2.00, m	-	1, 2, 4, 5
4	65.2	5.00, m	H-7	-
5	46.2	3.12, dd (18.1, 11.0); 2.93, dd (18.1, 4.8)	-	3, 4, 6
6	211.0	-	-	-
7	75.2	4.57, dd (7.7, 3.7)	H-4, H-9	8, 9
8	39.3	2.58, m; 2.41, m	-	6, 7, 9, 10
9	73.1	5.27, m	H-7	1, 7
10	34.3	1.81, m; 1.70, m	-	8, 9, 11, 12
11	25.8	1.27, m	-	10, 12, 13
12	31.8	1.23–1.09, m	-	-
13	22.7	1.21–1.11, m	-	-
14	14.1	0.76, t (6.6)	-	12, 13

^a^ Recorded at 100 MHz; ^b^ Recorded at 400 MHz.

### 2.2. Effects of Penicillinolide A *(1)* on the Expression of Pro-Inflammatory Proteins and Production of Pro-Inflammatory Cytokines in Murine Peritoneal Macrophages Stimulated with LPS

**Figure 4:**
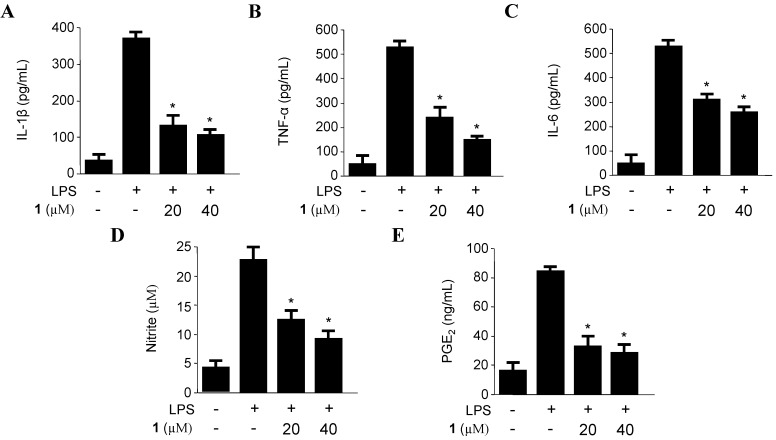
The effects of penicillinolide A (**1**) on the production of IL-1β (**A**); TNF-α (**B**); IL-6 (**C**); nitrite (**D**) and PGE_2_ (**E**) in murine peritoneal macrophages stimulated with LPS. Murine peritoneal macrophages were pre-treated for 12 h at the indicated concentrations of **1**, and stimulated for 18 h with LPS (500 ng/mL). The concentration of IL-1β, TNF-α, IL-6, NO and PGE_2_ were determined as described in the Experimental Section. The data represent the mean values ± SD of three experiments. * *p* < 0.05 compared to the group treated with LPS.

**Figure 5:**
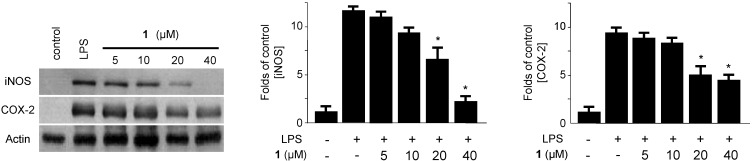
The effects of penicillinolide A (**1**) on the protein expression of iNOS and COX-2 in murine peritoneal macrophages stimulated with LPS. Murine peritoneal macrophages were pre-treated for 12 h at the indicated concentrations of **1**, and stimulated for 18 h with LPS (500 ng/mL). Western blot analysis was performed, and representative blots from three independent experiments are shown.

The effects of compounds on the LPS-induced IL-1β, TNF-α, IL-6, iNOS-derived NO and COX-2-derived PGE_2_ productions were examined by ELISA, in which macrophages were pre-incubated with **1** for 12 h before being stimulated with LPS (500 ng/mL) for 18 h in the presence or absence of non-cytotoxic concentrations of **1** (Supplementary Figure S1). Penicillinolide A (**1**) decreased the IL-1β, TNF-α, IL-6, NO, and PGE_2_ production in a concentration-dependent manner, with IC_50_ values of 8.63 µM, 11.32 µM, 20.92 μM, 20.47 μM, and 17.54 μM, respectively (Figure 4). To investigate the effects of **1** on iNOS and COX-2 expression in LPS-stimulated macrophages, murine peritoneal macrophages were stimulated with LPS in the presence or absence of **1** at non-cytotoxic concentrations (5–40 µM). Pre-treatment of the macrophages with **1** for 12 h resulted in a decrease in iNOS and COX-2 expression (Figure 5).

### 2.3. Effects of Penicillinolide A *(1)* on the Protein Expression Levels of IκB-α Phosphorylation and Degradation as well as NF-κB Translocation and DNA Binding Activity in Murine Peritoneal Macrophages

**Figure 6:**
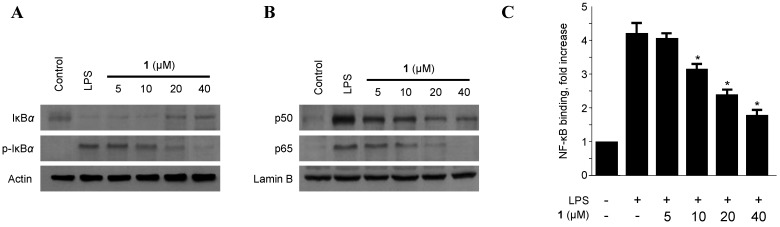
The effects of penicillinolide A (**1**) on the level of IκB-α phosphorylation, degradation of IκB-α (**A**); NF-κB p65 and p50 translocation (**B**); and NF-κB DNA binding activity (**C**) in murine peritoneal macrophages. Murine peritoneal macrophages were pre-treated for 12 h at the indicated concentrations of **1**, and stimulated for 1 h with LPS (500 ng/mL). Western blot analysis of IκB-α and p-IκB-α in the cytoplasm and NF-κB in the nucleus (**A**,**B**) were performed. A commercially available NF-κB ELISA (Active Motif) was used to test the nuclear extracts and to determine the degree of NF-κB binding (**C**). The data represent the mean values ± SD of three experiments. * *p* < 0.05 compared to the group treated with LPS.

The macrophage-triggering immunogen LPS, a component of the outer membrane of bacteria [28,29], activates the inhibitor of NF-κB (I-κB kinase), which in turn phosphorylates two specific serine residues on the I-κB protein. Phospho-IκB is then, after ubiquitination, degraded by the proteasome, which unmasks the nuclear localization signal of NF-κB, ultimately leading the way for its nuclear translocation and binding to target gene promoters, thereby up-regulating various inflammatory mediators such as TNF-α, IL-1β, IL-6, NO, PGE_2_, iNOS, and COX-2 [30,31]. Therefore, the phosphorylation and degradation of IκB-α (an inhibitor of NF-κB nuclear translocation) were evaluated as a next step to elucidate the mechanisms by which penicillinolide A (**1**) suppresses the production of LPS-induced pro-inflammatory enzymes and mediators. As shown in Figure 6, IκB-α in murine peritoneal macrophages was degraded after LPS treatment (500 ng/mL for 1 h). However, pre-treatment of **1** for 12 h, at concentrations ranging from 5 to 40 μM, markedly inhibited LPS-induced phosphorylation and degradation of IκB-α (Figure 6A), thereby preventing NF-κB (p50 and p65) translocation into the nucleus. The protein expression levels of nuclear p50 and p65 increased after treatment with LPS for 1h. However, this response was gradually inhibited by the treatment with **1** in a dose-dependent manner (Figure 6B). In addition, macrophages treated with LPS for 30 min showed an approximately three-fold increase in NF-κB DNA-binding activity as compared to controls. However, penicillinolide A (**1**) impaired this activity in a concentration-dependent manner (Figure 6C).

### 2.4. Effect of Penicillinolide *(1)* on HO-1 Expression via Nuclear Translocation of Nrf2 in Murine Peritoneal Macrophages

**Figure 7:**
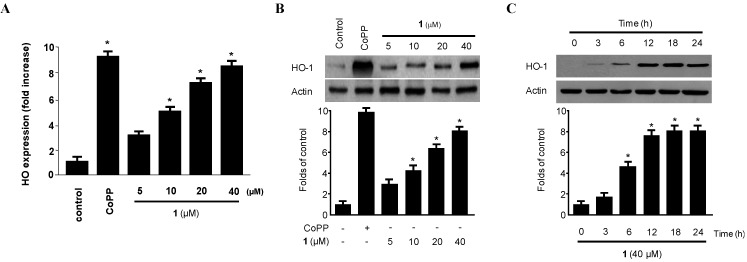
The effect of penicillinolide A (**1**) on the mRNA and protein expression of HO-1 in murine peritoneal macrophages. Murine peritoneal macrophages were incubated for 12 h with the indicated concentrations of **1**. HO-1 mRNA expression levels (**A**) were determined by real-time PCR. Western blot analyses showed concentration-dependent (**B**) and time-dependent (**C**) HO-1 protein expression. Representative blots from three independent experiments are shown. The data represent the mean ± SD of three experiments. * *p* < 0.05 compared to the control group.

HO-1 and its by-products suppress pro-inflammatory cytokine and chemokine production and promote anti-inflammatory responses through inhibition of NF-κB pathways in activated macrophages [4,5,6,7,8,9,10,11,32]. Therefore, we examined whether non-cytotoxic concentrations (5–40 µM) of penicillinolide A (**1**) affected HO-1 mRNA and protein expression in macrophages. As shown in Figure 7, penicillinolide A (**1**) significantly increased HO-1 mRNA (Figure 7A) and protein (Figure 7B) levels in a concentration-dependent manner, with a maximal value observed at 40 µM. The HO-1 inducer, cobalt protoporphyrin (CoPP), used as a positive control, increased HO-1 protein expression at a concentration of 20 μM. Treatment with **1** at 40 µM induced evident HO-1 expression at 6 h, peaked at around 18 h, and diminished after 24 h (Figure 7C).

**Figure 8:**
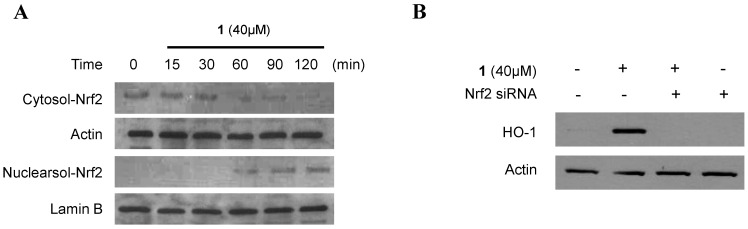
The effects of penicillinolide A (**1**) on the nuclear translocation of Nrf2 (**A**) and Nrf2-mediated HO-1 expression (**B**) in murine peritoneal macrophages. Murine peritoneal macrophages were treated with 40 μM of **1** for 15, 30, 60, 90 and 120 min. The nuclei were fractionated from the cytosol using PER-mammalian protein extraction buffer (**A**). Macrophages were transiently transfected with Nrf2 siRNA, and then were treated with 40 μM of **1** for 12 h (**B**). Western blot analysis for HO-1 expression and Nrf2 translocation was performed, as described in the Experimental Section, and the representative blots from three independent experiments are shown.

When cells are subjected to a variety of inflammatory conditions, they typically respond by inducing a coordinated expression of genes encoding the set of phase II detoxifying enzymes, principally involved in activation of transcription factors such as Nrf2 [33,34]. The Nrf2-mediated regulation of cellular anti-inflammatory responses plays a pivotal role in the defense against inflammatory conditions. In addition, Nrf2 plays a critical part in basal activity and coordinated induction of genes, encoding numerous antioxidant and phase II detoxifying enzymes and related proteins, such as HO-1 [33,34]. Therefore, we investigated whether treatment with **1** induces the translocation of Nrf2 to the nuclei in murine peritoneal macrophages. Macrophages were treated with 40 μM of **1** for 15, 30, 60, 90, and 120 min, and the level of Nrf2 protein was determined using Western blot analysis. Western blot analysis of the nuclear fraction of penicillinolide A-treated macrophages showed a gradual increase in Nrf2 levels, whereas the cytoplasmic fractions showed a concomitant decrease (Figure 8A). In addition, the role of Nrf2 in HO-1 expression by **1** was studied using siRNA against Nrf2. Macrophages were transiently transfected with siRNA Nrf2, and then were treated with 40 μM of **1** for 12 h. As shown in Figure 8B, transient transfection with Nrf2 siRNA completely abolished HO-1 expression by **1**, which suggested that penicillinolide A (**1**) was associated with HO-1 expression via Nrf2 signaling pathways.

### 2.5. Effects of SnPP on the Inhibition of Production of Pro-Inflammatory Mediators via Pre-Treatment of Penicillinolide A *(1)* in LPS-Stimulated Murine Peritoneal Macrophages

**Figure 9:**
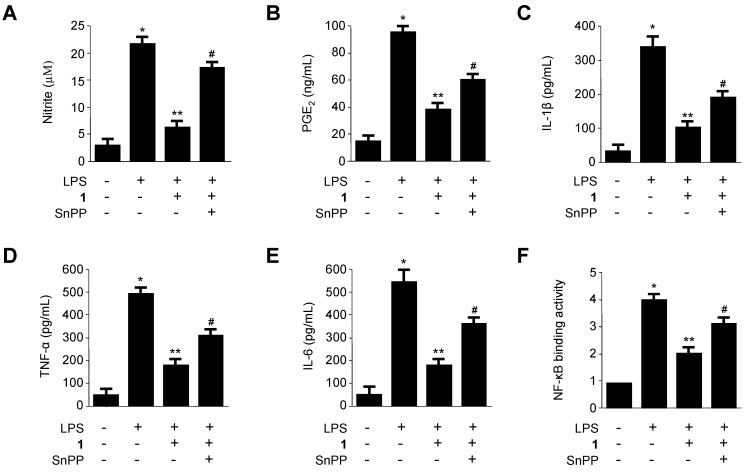
The effects of SnPP on the inhibition of nitrite, PGE_2_, TNF-α, IL-1β, and IL-6 production, and NF-κB DNA-binding activity by pre-treatment of **1** in LPS-stimulated murine peritoneal macrophages. Murine peritoneal macrophages were pre-treated for 12 h with **1** (40 μM), in the presence or absence of SnPP (50 μM) and stimulated with LPS (500 ng/mL) for 18 h (A–E) or 1 h (F). The concentrations of nitrite (**A**); PGE_2_ (**B**); TNF-α (**C**); IL-1β (**D**); and IL-6 (**E**) and nuclear NF-κB DNA-binding activity (**F**) were investigated as described in the Experimental Section. The data represent the mean ± SD of three experiments. * *p* < 0.05 compared to the control group; ** *p* < 0.05 compared to the group treated with LPS alone; # *p* < 0.05 compared to the group treated with **1** and LPS.

On the basis of our findings that penicillinolide A (**1**) markedly inhibited the production of LPS-induced pro-inflammatory enzymes and pro-inflammatory cytokines in the NF-κB signaling pathway (Figure 4, Figure 5 and Figure 6), and that penicillinolide A (**1**) induced the HO-1 expression via the Nrf2 pathway (Figure 7 and Figure 8), it was suggested that the up-regulation of HO-1 induced by penicillinolide A (**1**) mediated the suppressive effect on pro-inflammatory mediators through the NF-κB signaling pathway. To confirm this, we investigated whether the effect of penicillinolide A (**1**) was reversed by pre-treatment with SnPP, an inhibitor of HO-1. Murine peritoneal macrophages were pre-treated with 40 μM of **1** for 12 h in the absence or presence of SnPP. The suppressive effects of **1** on LPS-stimulated production of NO, PGE_2_, TNF-α, IL-1β, and IL-6 and NF-κB DNA-binding activity were partially reversed by SnPP (Figure 9). Therefore, these results suggest that induction of HO-1 by **1** partially contributes to the inhibitory effect of pro-inflammatory mediators through a NF-κB pathway.

## 3. Experimental Section

### 3.1.General Experimental Procedures and Materials

Optical rotations were recorded on a Perkin Elmer 341 digital polarimeter. UV spectra were recorded on a Biochrom 1300 UV/Visible spectrophotometer. The Fourier transform infrared (FTIR) spectrum was measured using a Nicolet 380 FT-IR spectrometer. NMR spectra (1D and 2D) were recorded in C_5_D_5_N or CDCl_3_ using a JEOL JNM ECP-400 spectrometer (400 MHz for ^1^H and 100 MHz for ^13^C), and chemical shifts were referenced relative to tetramethylsilane (δ_H_/δ_C_ = 0). HSQC and HMBC experiments were optimized for ^1^*J*_CH_ = 140 Hz and *^n^J*_CH_ = 8 Hz, respectively. ESIMS data were obtained using a Q-TOF micro LC-MS/MS instrument (Waters) at Korea University, Seoul, Korea. Solvents for extractions and flash column chromatography were reagent grade and used without further purification. Solvents used for HPLC were analytical grade. Flash column chromatography was performed using YMC octadecyl-functionalized silica gel (C_18_). HPLC separations were performed on a prep-C_18_ column (21.2 × 150 mm; 5-µm particle size) with a flow rate of 5 mL/min. Compounds were simultaneously detected by UV absorption at 210 nm and Evaporative Light Scattering Detector (ELSD).

Dulbecco’s modified Eagle’s medium (DMEM), fetal bovine serum (FBS), and other tissue culture reagents were purchased from Gibco BRL Co. (Grand Island, NY, USA). Tin protoporphyrin IX (SnPP IX), an inhibitor of HO activity, was obtained from Porphyrin Products (Logan, UT, USA). TG was purchased from BD Pharmingen (San Diego, CA, USA). All other chemicals were obtained from Sigma Chemical Co. (St. Louis, MO, USA) unless stated otherwise. Primary antibodies, including those raised against HO-1, COX-2, iNOS, IκB-α, p-IκB-α, and p65, and the appropriate secondary antibodies used for western blotting analysis were purchased from Santa Cruz Biotechnology (Santa Cruz, CA, USA). Enzyme-linked immunosorbent assay (ELISA) kits for PGE_2_, TNF-α, and IL-1β were purchased from R&D Systems (Minneapolis, MN, USA).

### 3.2. Specimen Collection and Identification of the Marine-Derived Fungus *Penicillium* sp. SF-5292

*Penicillium* sp. SF-5292 (deposited at the College of Medical and Life Sciences fungal strain repository, Silla University) was isolated from an unidentified organism belonging to Bryozoa that was manually collected using scuba equipment off the shores of Jeju Island in February 2009. The sample was stored in a sterile plastic bag and transported to the laboratory, where it was kept frozen until further processing. The sample was diluted 10 times using sterile seawater. The ground sample was diluted 10-fold using sterile seawater. One mL of the diluted sample was processed utilizing the spread plate method in potato dextrose agar (PDA) medium containing 3% NaCl. The plate was incubated at 25 °C for 14 days. After purifying the isolates several times, the final pure cultures were selected and preserved at −70 °C. This fungus was identified based on the analysis of the ribosomal RNA (rRNA) sequences. A GenBank search with the 28S rRNA gene of SF-5292 (isolation No. JF-34, GenBank accession number JN986843) indicated *Penicillium expansum* (JN938952), *P. solitum* (JN642222), *P. aurantiogriseum* (JN938945) and *P. polonicum* (JN938933) as the closest matches showing sequence identities of 99.89%, 99.89%, 99.77% and 99.77%, respectively. Therefore, the marine-derived fungal strain SF-5292 was characterized as *Penicillium* sp.

### 3.3. Fermentation, Extraction and Isolation of Penicillinolide A *(1)* from *Penicillium* sp. SF-5292

The fungal strain was cultured on 60 petri-dish plates (90 mm), each containing 20 mL of PDA [0.4% (w/v) potato Starch, 2% (w/v) dextrose, 3% (w/v) NaCl, 1.5% (w/v) agar]. Plates were individually inoculated with 2 mL seed cultures of the fungal strain. Plate cultures were incubated at 25 °C for a period of 14 days. Extraction of the agar media with MEK (4 × 1 L) provided an organic phase, which was then concentrated *in vacuo* to yield 1.0 g of extract. The MEK extract was subjected to C_18_ flash column chromatography (5 × 40 cm), eluting with a stepwise gradient of 20%, 40%, 60%, 80%, and 100% (v/v) MeOH in H_2_O (400 mL each). The fractions eluted at 80% MeOH (140.7 mg) were combined and purified by semi-preparative reversed-phase HPLC eluting with a gradient from 40% to 100% MeOH in H_2_O (0.1% formic acid) over 60 min, then 100% MeOH for 20 min, to yield penicillinolide A (**1**, 13.4 mg, *t*_R_ = 31.0 min).

Penicillinolide A (**1**): White solid; [α]^25^_D_ +50 (*c* 0.38, MeOH); FT-IR ν_max_ 3393, 2956, 2932, 2859, 1717, 1440, 1350, 1250, 1128, 1100, 1043, 963, 925 cm^−1^; ^1^H and ^13^C NMR data, Table 1; HRESIMS *m/z* 295.1517 [M + Na]^+^ (calcd for C_14_H_24_O_5_Na, 295.1521).

### 3.4. Preparation of Mosher Esters of Penicillinolide A *(1)*

Compound **1** (2 mg) was treated with (*R*)-(-)- and (*S*)-(+)-α-methoxy-α-(trifluoromethyl)phenylacetyl chloride (15 µL) in CH_2_Cl_2_ (400 µL) in the presence of 4-dimethylaminopyridine for 24 h at room temperature. The reaction was monitored by silica gel TLC and stopped when the original spot had disappeared. The reaction product was purified by preparative silica gel TLC using *n*-hexane-acetone (2:1) as eluent to give (*S*)-(**1a**) and (*R*)-MTPA esters (**1b**), respectively. The ^1^H NMR spectra of the esters were recorded in CDCl_3_ and the assignments were done by ^1^H–^1^H COSY spectra.

#### 3.4.1. Selected 1H NMR (CDCl3, 400 MHz) Data for **1a**

δ_H_ 7.35-7.52 (5H, Ph), 5.77 (1H, d, *J* = 10.8 Hz, H-4), 4.93 (2H, H-7 and H-9), 3.57 (3H, s, OCH_3_), 2.95 (1H, dd, *J* = 4.0, 18.0 Hz, H-5b), 2.77 (1H, dd, *J* = 11.2, 18.0 Hz, H-5a), 2.29 (1H, m, H-8a), 2.24 (1H, m, H-3a), 2.14 (1H, m, H-8b), 1.83 (1H, m, H-3b), 1.42 (2H, m, H_2_-10), 0.76 (3H, t, *J* = 6.8 Hz, H_3_-14).

#### 3.4.2. Selected 1H NMR (CDCl3, 400 MHz) Data for **1b**

δ_H_ 7.33-7.45 (5H, Ph), 5.77 (1H, d, *J* = 10.8 Hz, H-4), 4.96 (2H, H-7 and H-9), 3.46 (3H, s, OCH_3_), 2.90 (1H, dd, *J* = 4.4, 18.0 Hz, H-5b), 2.69 (1H, dd, *J* = 11.2, 18.0 Hz, H-5a), 2.36 (1H, m, H-8a), 2.30 (1H, m, H-3a), 2.18 (1H, m, H-8b), 1.87 (1H, m, H-3b), 1.43 (2H, m, H_2_-10), 0.78 (3H, t, *J* = 6.8 Hz, H_3_-14).

### 3.5. Peritoneal Macrophage Cultures and Cell Viability Assay

C57BL/6 mice were purchased from Orient Bio Co. (Sungnam, Kyung-Kido, Korea). TG-elicited peritoneal macrophages were harvested 4 days after intraperitoneal (i.p.) injection of 3 mL of TG [35]. Peritoneal lavage was performed using 8 mL of Hanks’ balanced salt solution (HBSS) containing 10 U/mL heparin. The cells were distributed in Roswell Park Memorial Institute (RPMI) medium supplemented with 10% heat-inactivated FBS, in 6-well tissue culture plates (5 × 10^6^ cells/mL). The effects of various experimental modulations on cell viability were evaluated according to mitochondrial reductase function by using an assay based on the reduction of tetrazolium salt 3-[4,5-dimethylthiazol-2-yl]-2,5-diphenyltetrazolium bromide (MTT) with formazan crystals [36].

### 3.6. Determination of Nitrite Production and PGE_2_, TNF-α, IL-1β and IL-6 Assays

The production of nitrite, a stable end product of NO oxidation, was used as a measure of iNOS activity. The nitrite present in the conditioned medium was determined using a method based on the Griess reaction [37]. The level of PGE_2_, TNF-α, IL-1β or IL-6 present in each sample was determined using a commercially available kit from R&D Systems [38]. The assay was performed according to the manufacturer’s instructions. Briefly, murine peritoneal macrophages were cultured in 24-well plates, pre-incubated for 12 h with different concentrations of compounds, and then stimulated for 18 h with LPS. The cell culture supernatants were then immediately collected after treatment and centrifuged at 13,000× *g* for 2 min to remove particulate matter. The medium was added to a 96-well plate pre-coated with affinity-purified PGE_2_-specific polyclonal antibodies or the medium was added to a 96-well plate pre-coated with affinity-purified polyclonal antibodies that were specific to mouse TNF-α, IL-1β or IL-6. An enzyme-linked polyclonal antibody specific for PGE_2_, mouse TNF-α, IL-1β or IL-6 was added to the wells for 20 h, followed by a final wash to remove any unbound antibody-enzyme reagent. A substrate solution was added, and the intensity of the color produced, which was measured at 450 nm (the correction wavelength was set at 540 nm or 570 nm), was proportional to the amount of PGE_2_, TNF-α, IL-1β or IL-6 present.

### 3.7. Preparation of Cytosolic and Nuclear Fractions

Murine peritoneal macrophages were homogenized (1:20, w:v) in PER-Mammalian Protein Extraction buffer (Pierce Biotechnology, Rockford, IL, USA) containing freshly added protease inhibitor cocktail I (EMD Biosciences, San Diego, CA, USA) and 1 mM phenylmethylsulfonyl fluoride (PMSF). The cytosolic fraction of the cells was prepared by centrifugation at 15,000× *g* for 10 min at 4 °C. Nuclear and cytoplasmic extracts of cells were prepared using NE-PER nuclear and cytoplasmic extraction reagents (Pierce Biotechnology), respectively.

### 3.8. Western Blot Analysis

Western blot analysis was performed by lysing the cells in 20 mM Tris-HCl buffer (pH 7.4) containing a protease inhibitor mixture (0.1 mM PMSF, 5 mg/mL aprotinin, 5 mg/mL pepstatin A, and 1 mg/mL chymostatin). The protein concentration was determined using a Lowry protein assay kit (P5626; Sigma Chemical Co., St Louis, MO, USA). An equal amount of protein for each sample was resolved using 12% sodium dodecyl sulfate-polyacrylamide gel electrophoresis (SDS-PAGE) and then electrophoretically transferred onto a Hybond enhanced chemiluminescence (ECL) nitrocellulose membrane (Bio-Rad, Hercules, CA, USA). The membrane was blocked with 5% skimmed milk and sequentially incubated with primary antibody (Santa Cruz Biotechnology) and horseradish peroxidase-conjugated secondary antibody followed by ECL detection (Amersham Pharmacia Biotech, Piscataway, NJ, USA).

### 3.9. Real-Time PCR

Total RNA was isolated from the cells by using Trizol (Invitrogen, Carlsbad, CA, USA), in accordance with the manufacturer’s recommendations, and quantified spectrophotometrically (at 260 nm). Total RNA (1 μg) was reverse-transcribed using the High Capacity RNA-to-cDNA kit (Applied Biosystems, Carlsbad, CA, USA). The cDNA was then amplified using the SYBR Premix Ex Taq kit (TaKaRa Bio Inc., Shiga, Japan) by using a StepOnePlus Real-Time PCR system (Applied Biosystems). Briefly, each 20 μL of reaction volume contained 10 μL of SYBR Green PCR Master Mix, 0.8 μM of each primer, and diethyl pyrocarbonate (DEPC)-treated water. The primer sequences were designed using PrimerQuest (Integrated DNA Technologies, Cambridge, MA, USA). The primer sequences were as follows: HO-1, forward 5′-CTCTTGGCTGGCTTCCTT-3′, reverse 5′-GGCTCCTTCCTCCTTTCC-3′, and glyceraldehyde 3-phosphate dehydrogenase (GAPDH), forward 5′-ACTTTGGTATCGTGGAAGGACT-3′, reverse 5′-GTAGAGGCAGGGATGATGTTCT-3. The optimal conditions for PCR amplification of the cDNA were established by following the manufacturer’s instructions. The data were analyzed using StepOne software (Applied Biosystems), and the cycle number at the linear amplification threshold (Ct) values for the endogenous control gene (GAPDH) and the target gene were recorded. Relative gene expression (target gene expression normalized to the expression of the endogenous control gene) was calculated using the comparative Ct method (2^−ΔΔCt^).

### 3.10. DNA-Binding Activity of NF-κB

The DNA-binding activity of NF-κB in the nuclear extracts was measured using the TransAM kit (Active Motif, Carlsbad, CA, USA) according to the manufacturer’s instructions.

### 3.11. Transfection of Nrf2 siRNA

Macrophages were transiently transfected with Nrf2 siRNA for 6 h by using LipofectAMINE 2000TM (Invitrogen, Grand Island, NY, USA), according to the manufacturer’s protocol, and recovered in fresh media containing 10% FBS for 24 h.

### 3.12. Statistical Analysis

The data were expressed as the mean ± standard deviation (SD) of at least three independent experiments. To compare three or more groups, one-way analysis of variance (ANOVA) followed by the Newman-Keuls *post hoc* test was used. Statistical analysis was performed using GraphPad Prism software, version 3.03 (GraphPad Software Inc., San Diego, CA, USA).

## 4. Conclusions

Chemical investigation of the marine-derived fungus *Penicillium* sp. SF-5292 afforded a new 10-membered lactone, named penicillinolide A (**1**). Penicillinolide A (**1**) is a new member of naturally occurring 10-membered lactones (nonenolides, decanolides), which showed a broad spectrum of bioactivities such as cytotoxic, phytotoxic, antimalarial, antimicrobial [39], and inhibition of cholesterol biosynthesis [40]. These various bioactivities together with the interesting structure of the medium-sized ring have led to a number of synthetic studies [39]. The structure of penicillinolide A (**1**) is most closely related to putaminoxins B and D, which have a pentyl side chain at the C-9 position of the 10-membered lactone ring [41]. In addition, phomolide D, which has a propyl side chain at the C-9 position of the lactone ring, shares similar structural features with penicillinolide A (**1**) [42]. Penicillinolide A (**1**), to the best of our knowledge, is the first member of the nonenolides that has an anti-inflammatory activity.

In the course of further pharmacological evaluation of penicillinolide A (**1**), it was shown that **1** suppressed the production of pro-inflammatory mediators such as NO, PGE_2_, TNF-α, IL-1β, and IL-6 via the inhibition of the NF-κB pathway. In addition the anti-inflammatory effect of **1** was shown to be associated with the Nrf2-related induction of anti-inflammatory HO-1 enzyme expression in LPS-induced murine peritoneal macrophages. Therefore, penicillinolide A (**1**) may be a potential therapeutic candidate for the treatment of inflammatory diseases.

## Supplementary Files

Supplementary File 1 Supplementary Information (PDF, 395 KB)
