# Supplementary material for: Penicillinolide A: A New Anti-Inflammatory Metabolite from the Marine Fungus Penicillium sp. SF-5292
Source: Mar Drugs. 2013 Nov 12;11(11):4510–26. doi: 10.3390/md11114510 (PMC3853742; doi:10.3390/md11114510)

Supplementary Information

Table of Contents

Figure S1. The effects of penicillinolide A (**1**) on cell viability in murine peritoneal macrophages.
Figure S2. ^{1}H NMR spectrum of penicillinolide A (**1**) in pyridine-d_5.
Figure S3. ^{13}C NMR spectrum of penicillinolide A (**1**) in pyridine-d_5.
Figure S4. HSQC data of penicillinolide A (**1**) in pyridine-d_5.
Figure S5. COSY data of penicillinolide A (**1**) in pyridine-d_5.
Figure S6. HMBC data of penicillinolide A (**1**) in pyridine-d_5.
Figure S7. NOESY data of penicillinolide A (**1**) in pyridine-d_5.
Figure S8. ^{1}H NMR spectrum of (*S*)-MTPA ester of penicillinolide A (**1**) in $CDCl_3$.
Figure S9. COSY data of (*S*)-MTPA ester of penicillinolide A (**1**) in $CDCl_3$.
Figure S10. ^{1}H NMR spectrum of (*R*)-MTPA ester of penicillinolide A (**1**) in $CDCl_3$.
Figure S11. COSY data of (*R*)-MTPA ester of penicillinolide A (**1**) in $CDCl_3$.

Figure S1. The effects of compounds on cell viability in murine peritoneal macrophages. Murine peritoneal macrophages were incubated for 48 h with various concentrations of each compound (1–50 μM). Cell viability was determined. The data represent the mean values ± SD of 3 experiments.

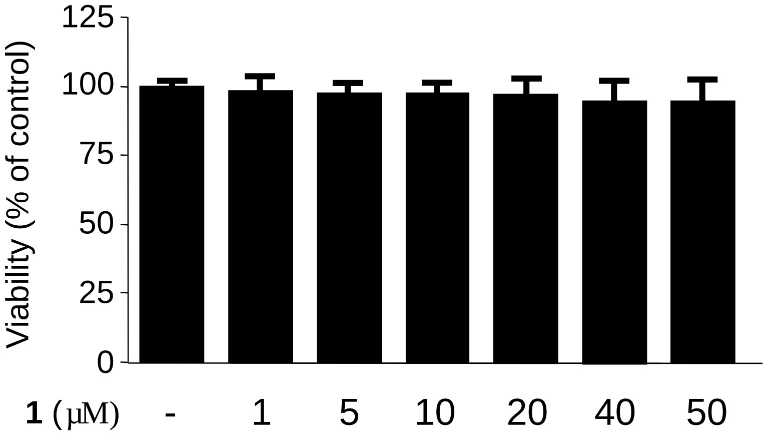

Figure S2. ^{1}H NMR spectrum of penicillinolide A (**1**) in pyridine-d_5.

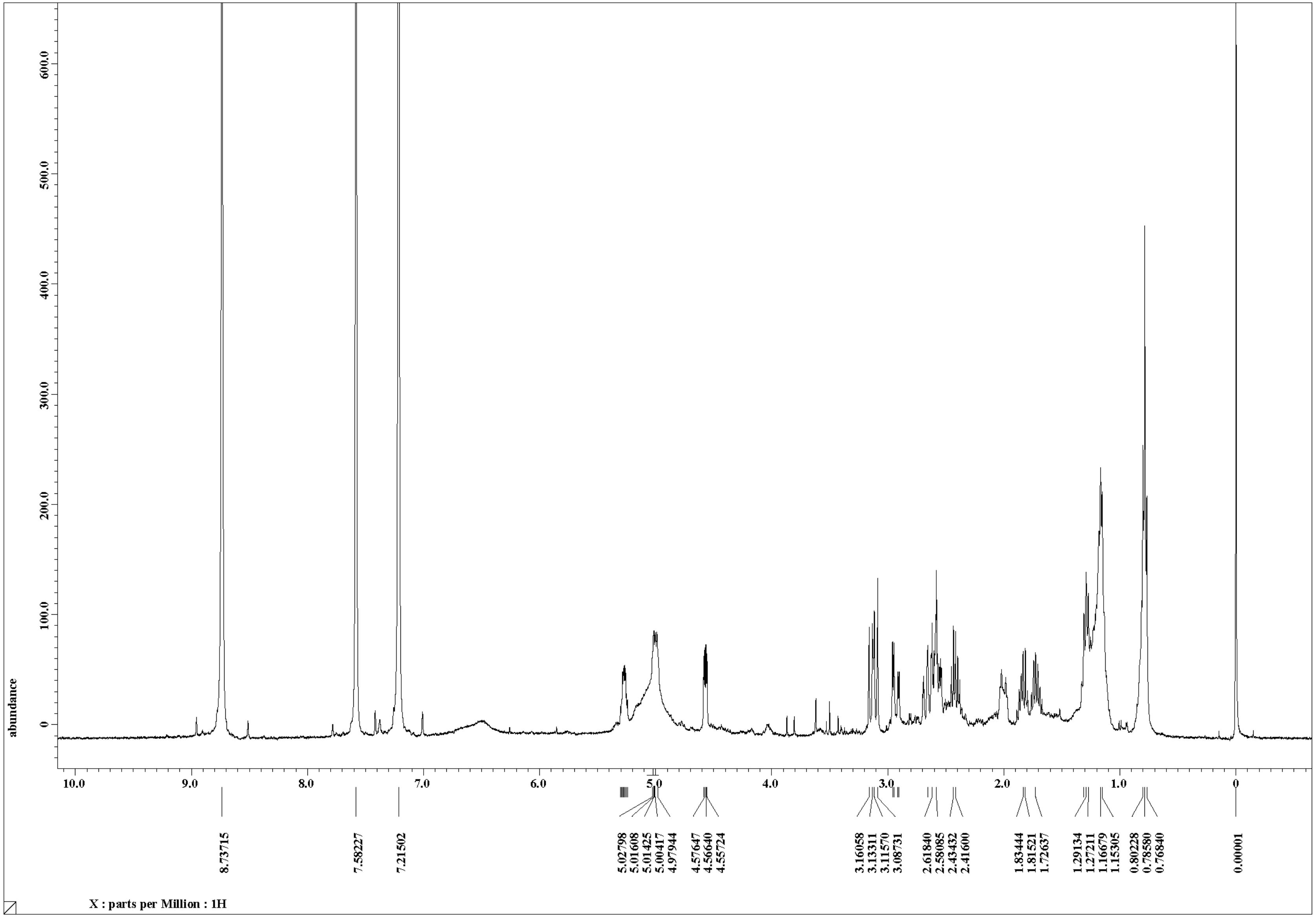

Figure S3. ^{13}C NMR spectrum of penicillinolide A (**1**) in pyridine-d_5.

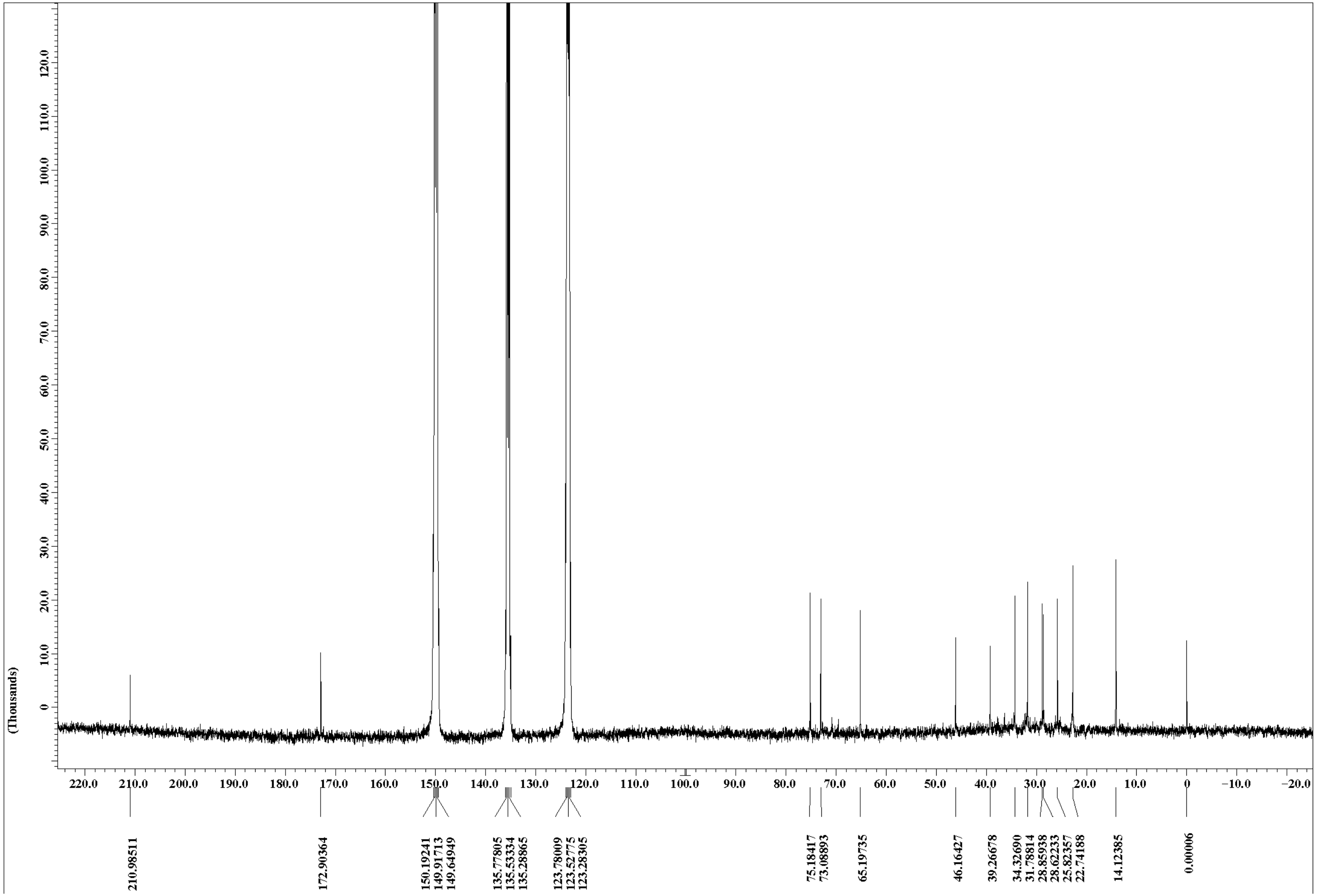

Figure S4. HSQC data of penicillinolide A (**1**) in pyridine-d_5.

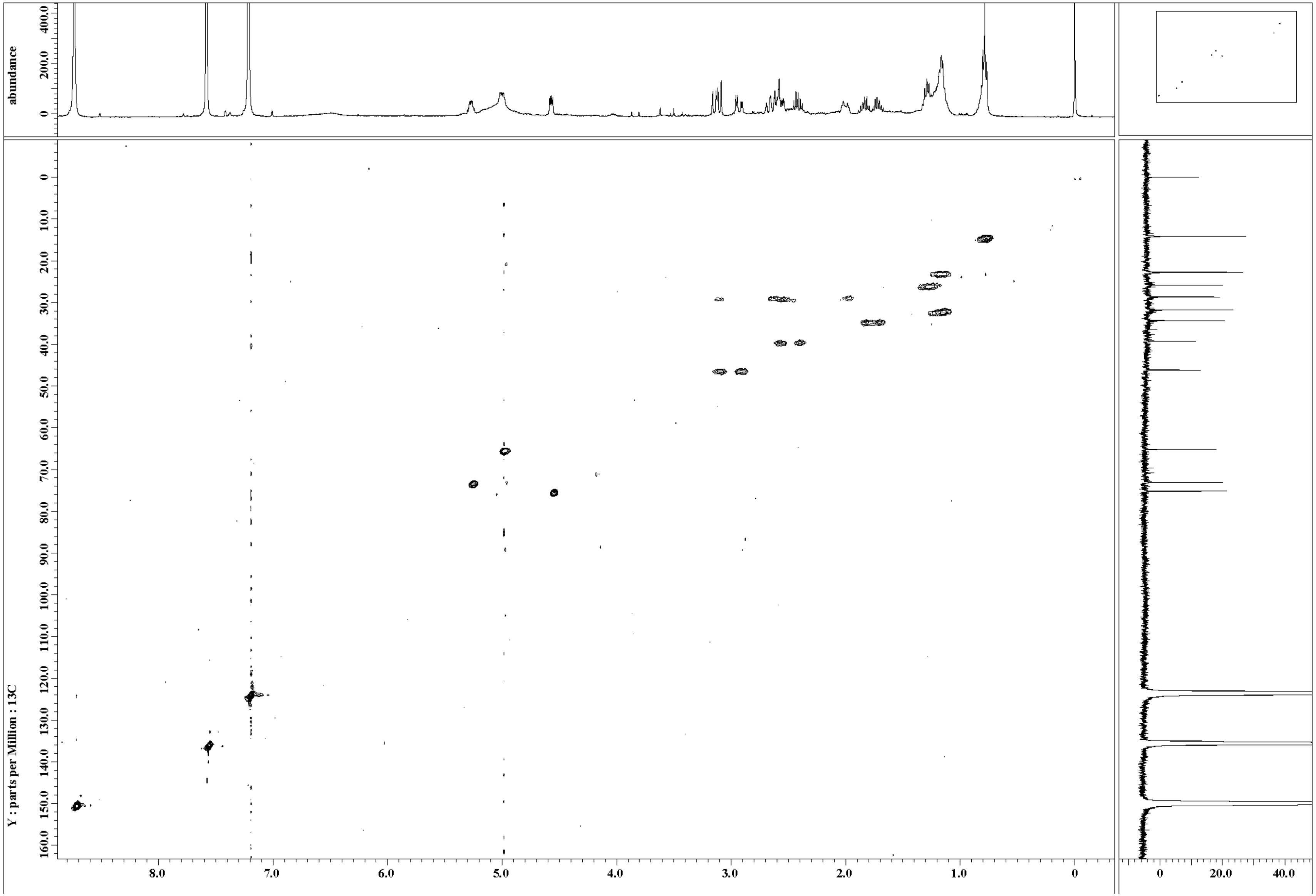

Figure S5. COSY data of penicillinolide A (**1**) in pyridine-d_5.

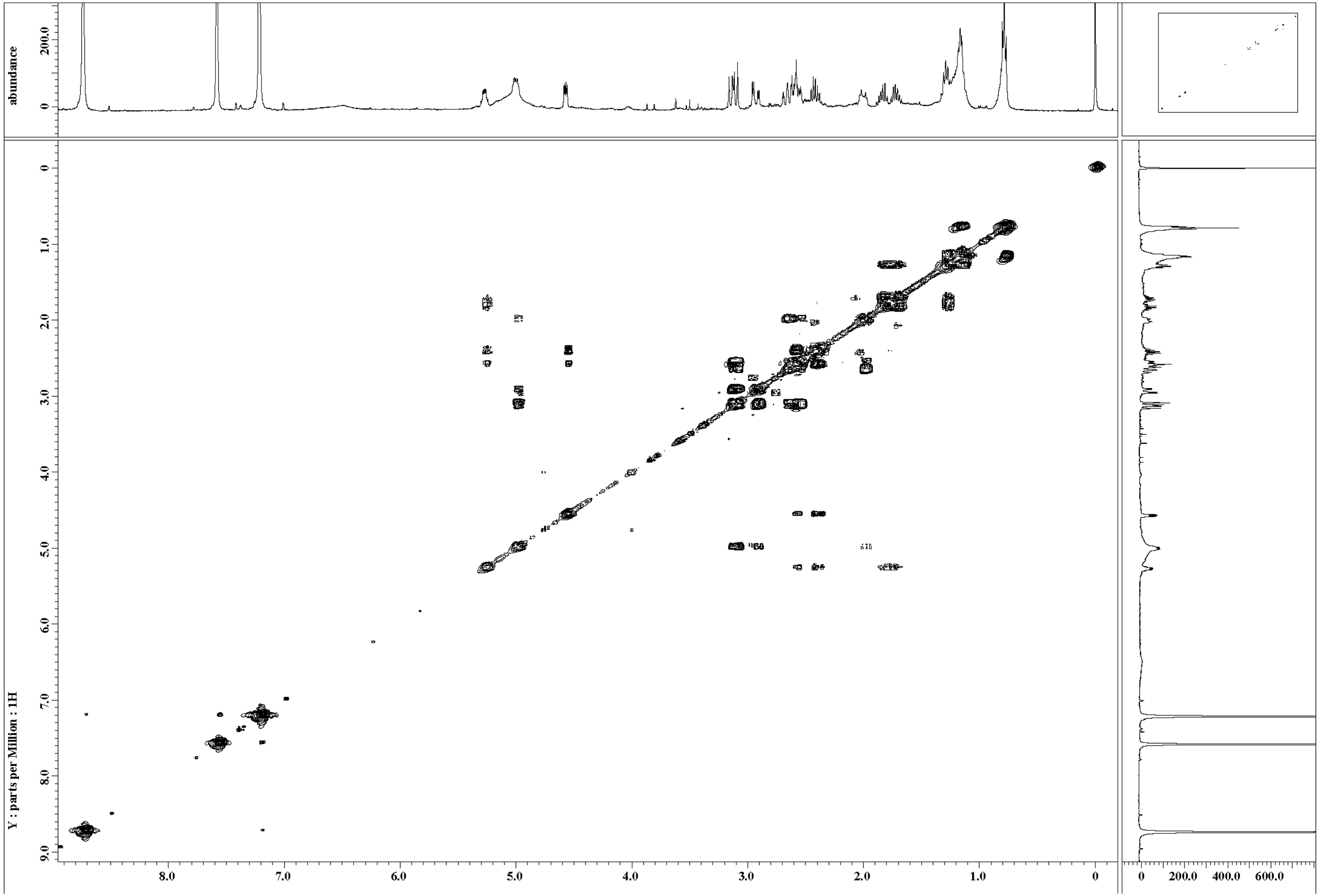

Figure S6. HMBC data of penicillinolide A (**1**) in pyridine-d_5.

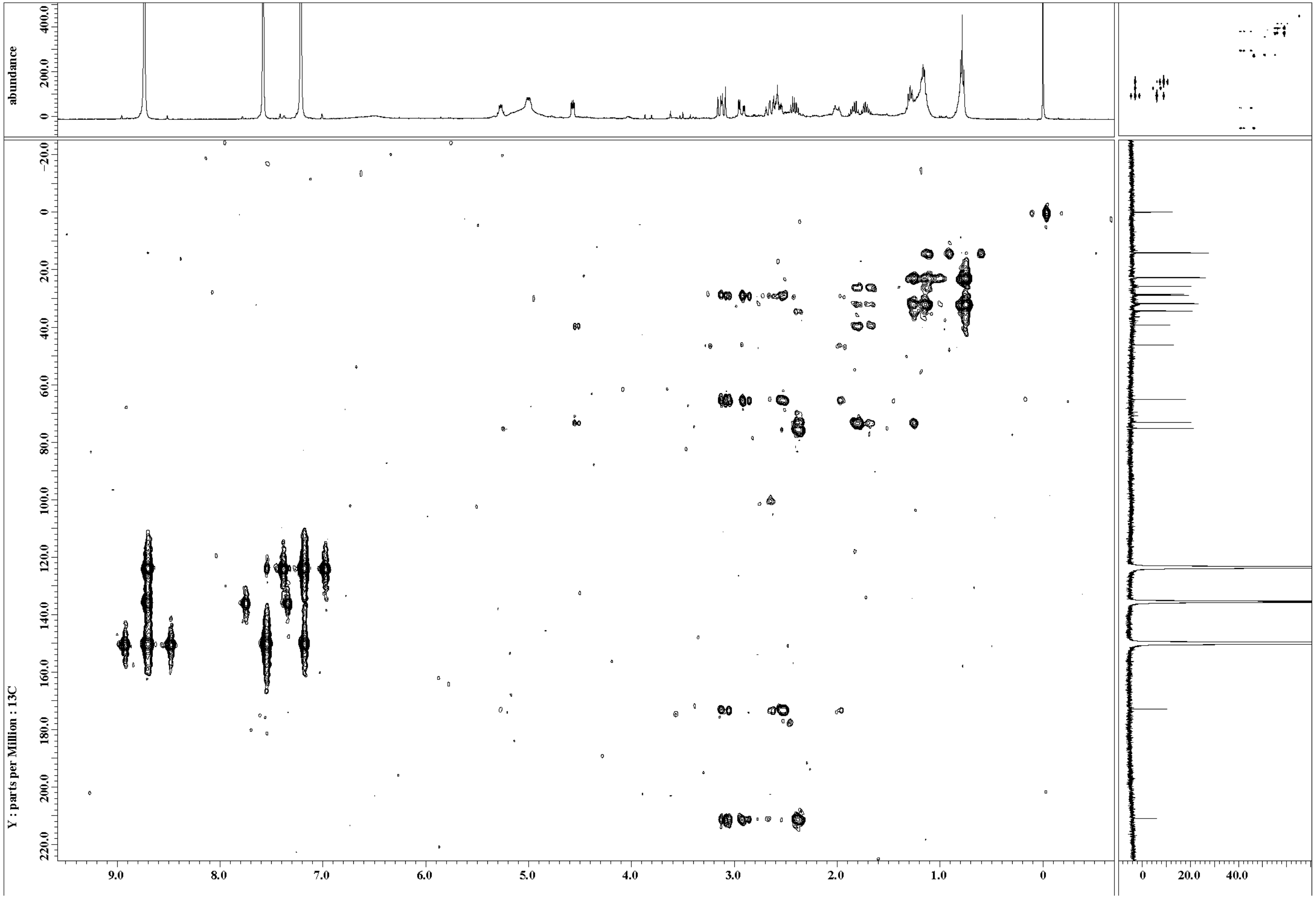

Figure S7. NOESY data of penicillinolide A (**1**) in pyridine-d_5.

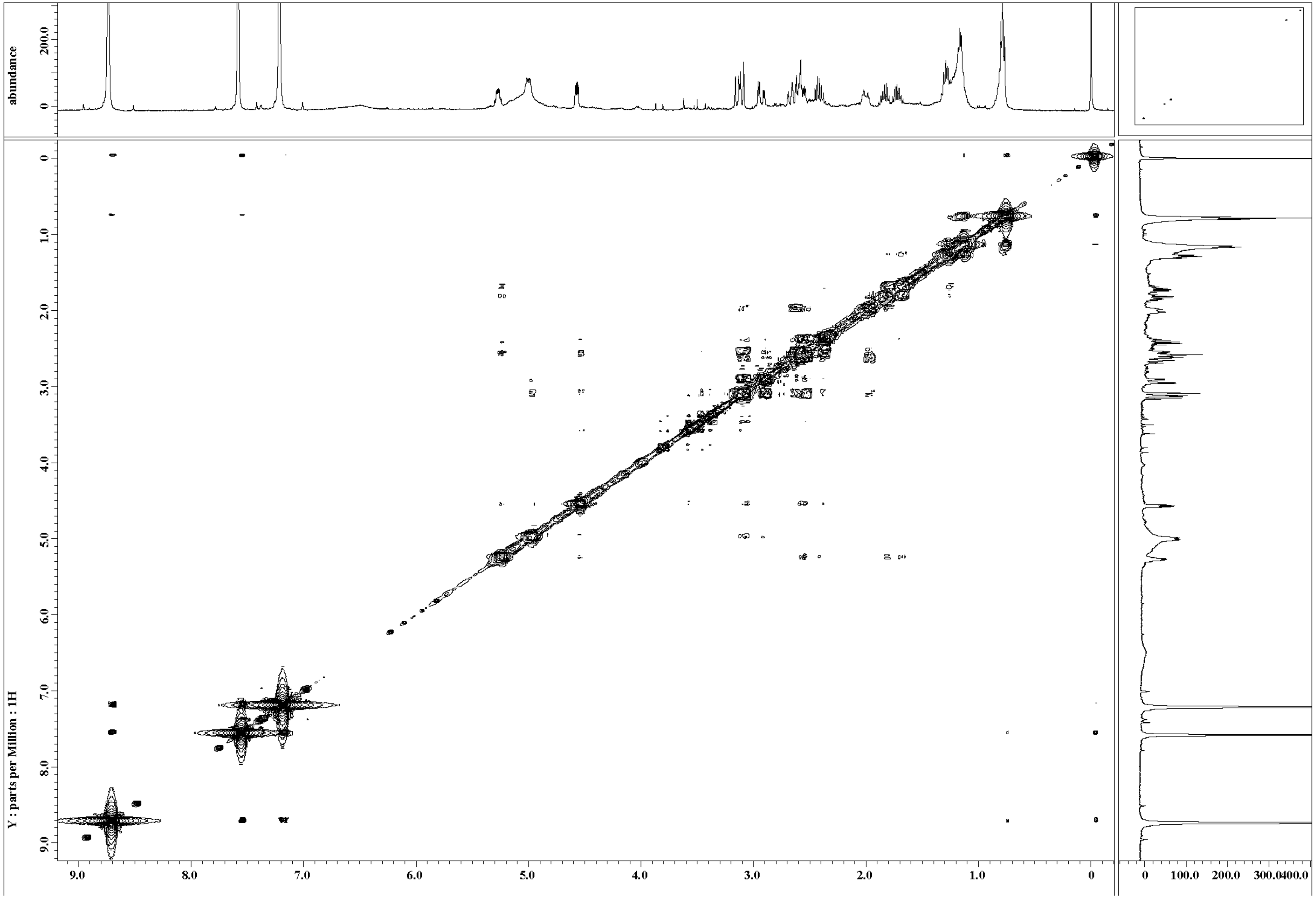

Figure S8. ^{1}H NMR spectrum of (*S*)-MTPA ester of penicillinolide A (**1**) in $CDCl_3$.

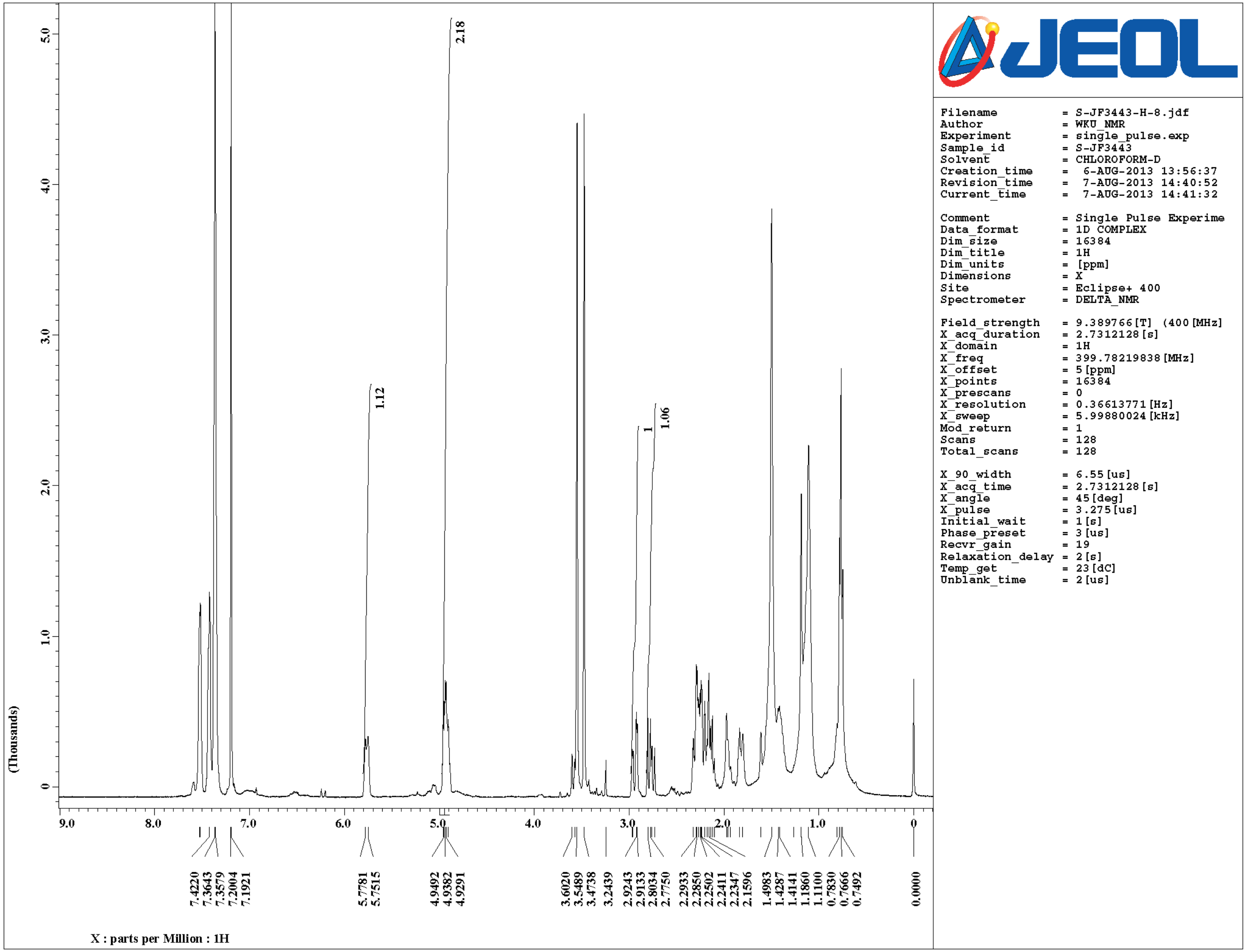

Figure S9. COSY data of (*S*)-MTPA ester of penicillinolide A (**1**) in $CDCl_3$.

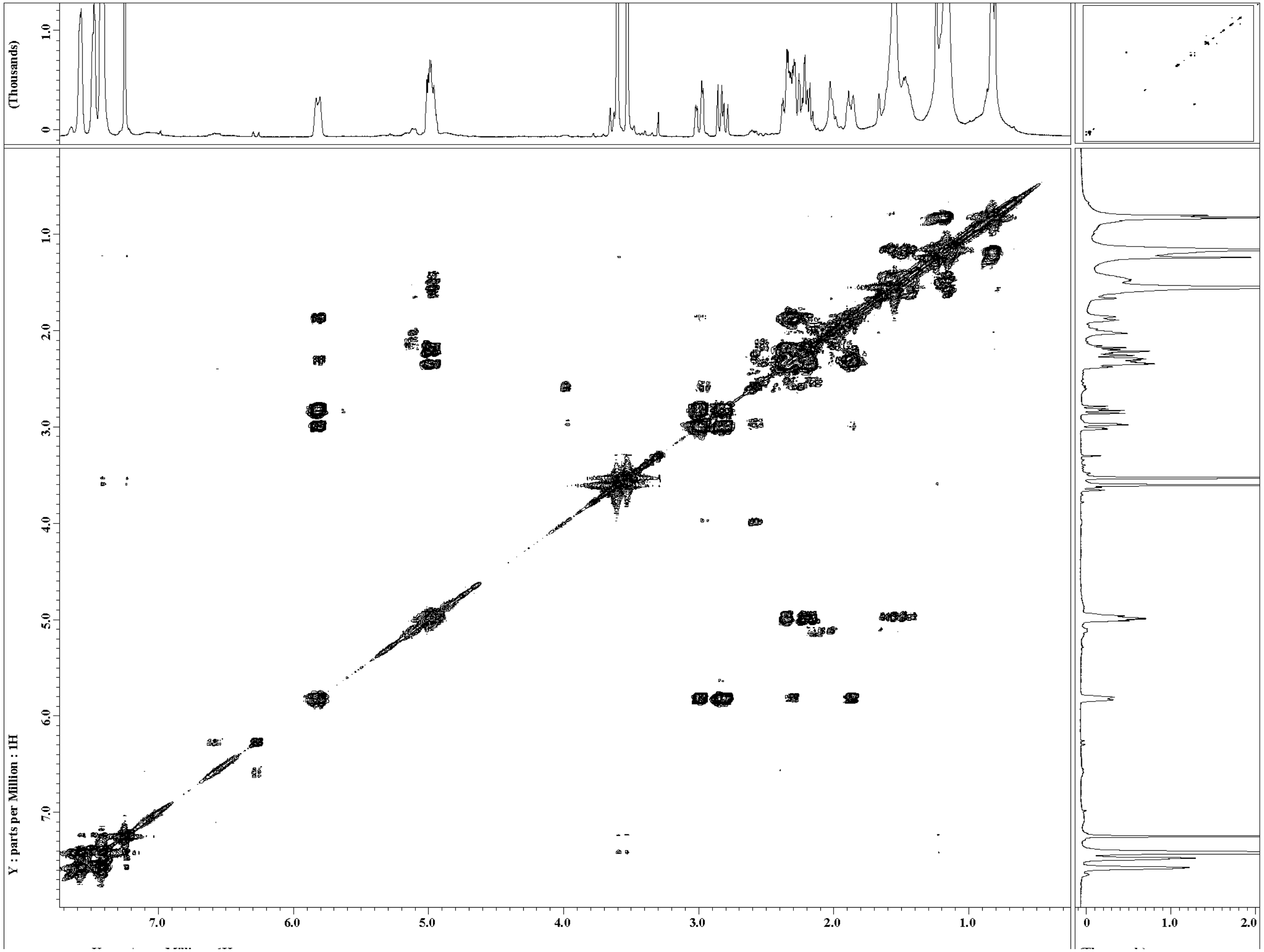

Figure S10. ^{1}H NMR spectrum of (*R*)-MTPA ester of penicillinolide A (**1**) in $CDCl_3$.

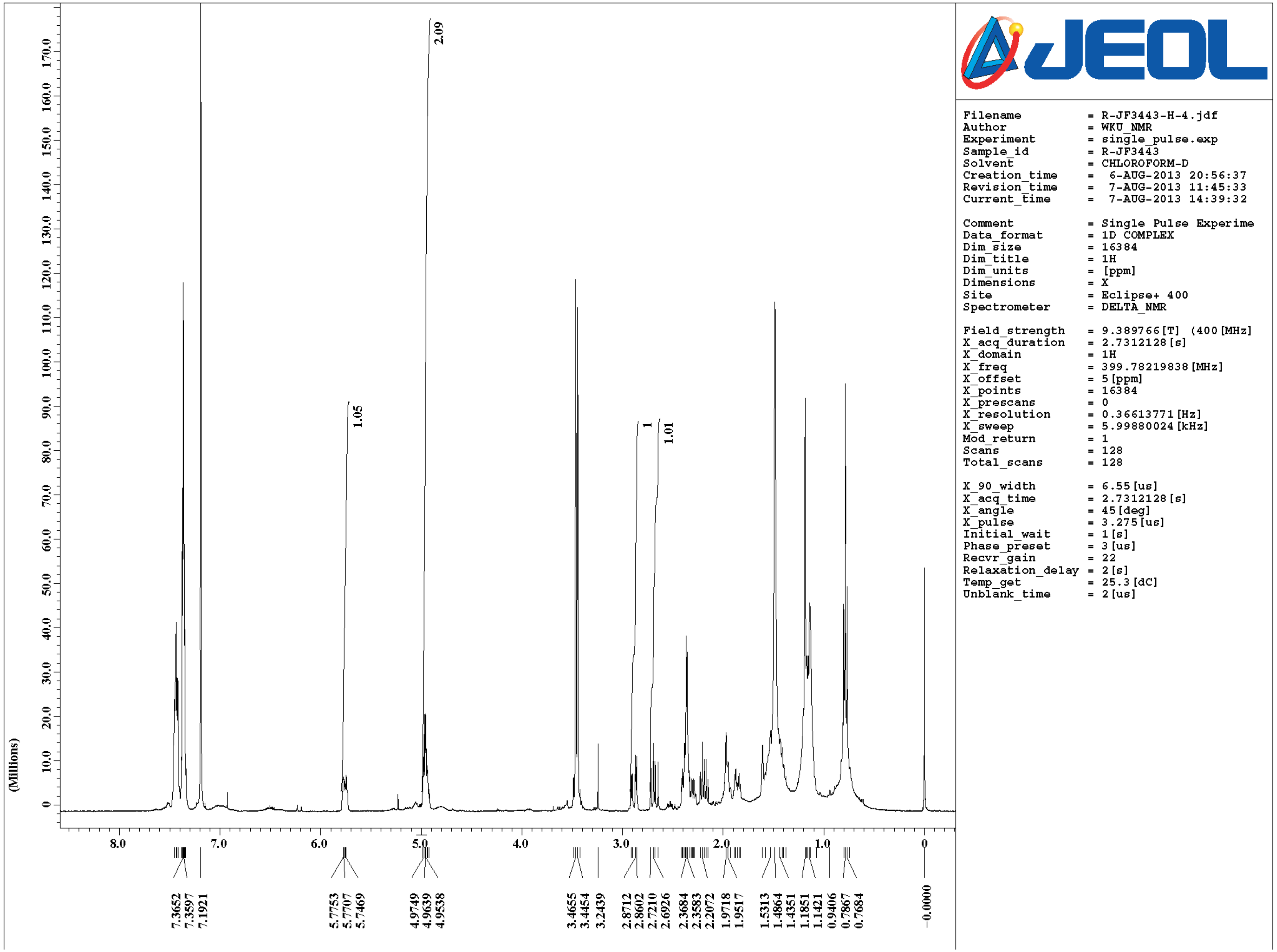

Figure S11. COSY data of (*R*)-MTPA ester of penicillinolide A (**1**) in $CDCl_3$.

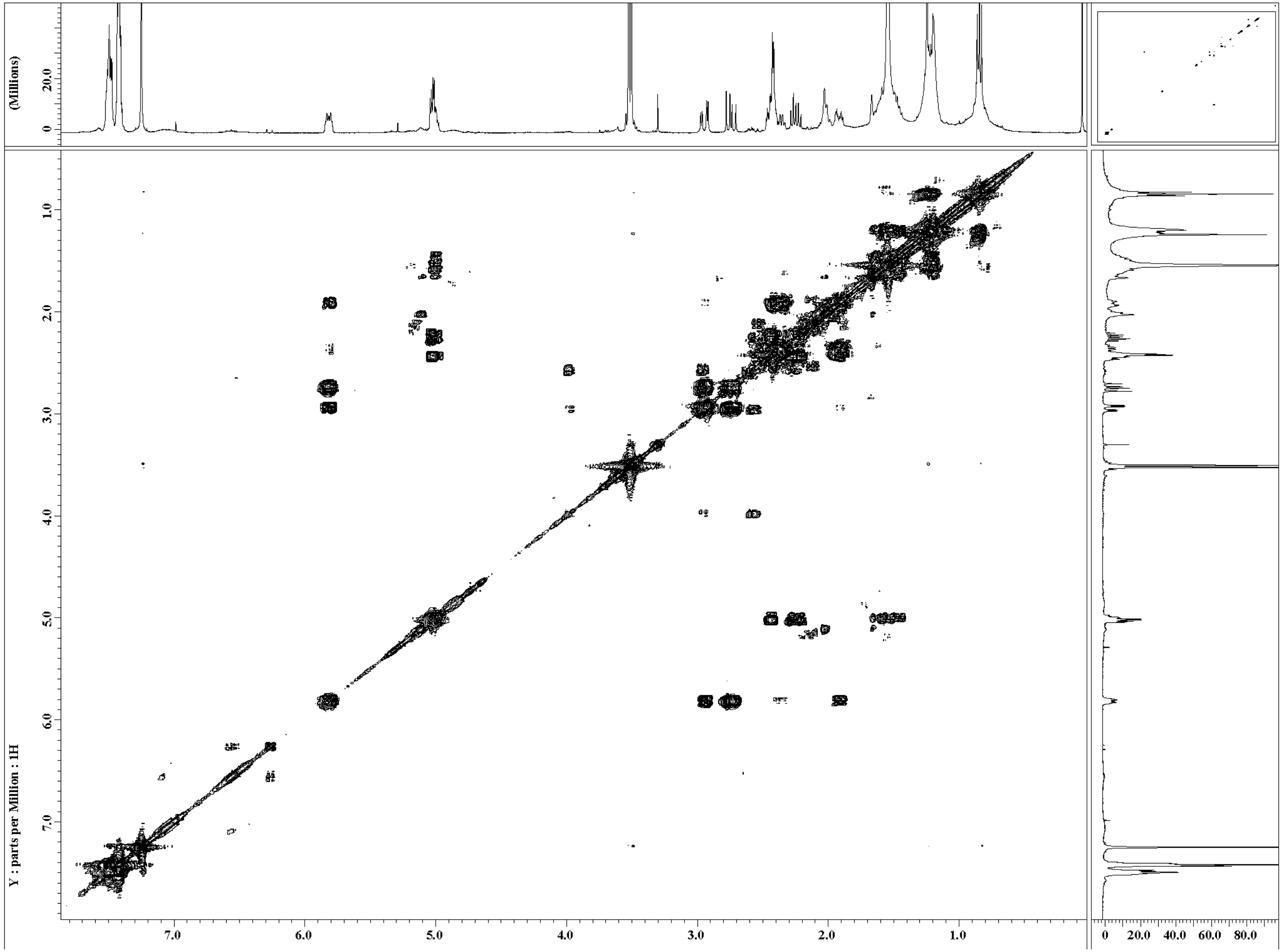

Supplement: Supplementary File 1 — Supplementary Information (PDF, 395 KB) [file marinedrugs-11-04510-s001.pdf]
